# Application of T‐Type Drainage Tube in Treating Large Deep Vaginal Hematoma Postpartum: A Technical Note

**DOI:** 10.1155/crog/4448042

**Published:** 2026-01-30

**Authors:** Chunxia Lin, Jie Lin, Jun Zhou, Jia Tang, Miaomiao Tang, Dandan Wu, Shiling Jiang, Hui Cheng, Li Feng, Zhaoping Zheng, Qingyu Tang, Yuan Ming

**Affiliations:** ^1^ Department of Obstetrics, West China Longquan Hospital Sichuan University/The First People′s Hospital of Longquanyi District Chengdu, Chengdu, Sichuan, China, cdlqyyy.com; ^2^ Department of Operating Room, West China Longquan Hospital Sichuan University/The First People′s Hospital of Longquanyi District Chengdu, Chengdu, Sichuan, China, cdlqyyy.com; ^3^ Department of Ultrasound Medicine, West China Longquan Hospital Sichuan University/The First People′s Hospital of Longquanyi District Chengdu, Chengdu, Sichuan, China, cdlqyyy.com

**Keywords:** deep vaginal wall hematoma, T-shaped drainage catheter, vaginal delivery, vaginal packing

## Abstract

**Background:**

Deep vaginal wall hematoma is a common vaginal delivery complication. Exploring effective treatments is key to improving clinical management and patient outcomes.

**Case:**

A patient with deep vaginal wall hematoma (admitted in November 2024) underwent hematoma removal, followed by “T”‐shaped drainage tube combined with vaginal packing gauze. The treatment achieved remarkable hemostasis; the patient recovered rapidly with well‐healed perineal wounds, shorter hospital stays, and low pain scores.

**Conclusions:**

Early detection and timely treatment of vaginal wall hematoma, combined with “T”‐shaped drainage tube drainage, avoid open abdominal hemostasis and related damage. This method is safe, effective, and worthy of clinical application.

## 1. Introduction

Postpartum hematoma refers to a hematoma that occurs in the soft birth canal (including the broad ligament, pelvic peritoneum, ischiorectal fossa, perineum, vagina, cervix, and lower uterus) during labor and within a few hours postpartum. The most common site of postpartum hematoma in clinical practice is the vagina, which is a form of postpartum hemorrhage [1]. Vaginal wall hematoma is a common complication that occurs during and after childbirth. Hidden bleeding is easily overlooked, and calculating the amount of bleeding is difficult. If not detected or properly treated in a timely manner, it can easily lead to secondary anemia, infection, or postpartum bleeding. In severe cases, it can cause hemorrhagic shock and even endanger life [2]. Therefore, obstetric medical staff need to attach great importance to the high‐risk factors that lead to the formation of vaginal hematomas in pregnant women. Early detection and treatment can prevent more serious postpartum complications. This article analyzes the treatment process of a postpartum woman with deep vaginal wall hematoma who was treated in our hospital on November 7, 2024. The successful experience of treatment is shared, and the treatment situation is reported as follows. Epidemiological studies have reported the prevalence of pelvic floor hematoma (including vaginal wall hematoma) after vaginal delivery, further confirming the clinical commonality of this complication [3].

## 2. Clinical Data

### 2.1. General

The patient, a 30‐year‐old female, was admitted on November 7, 2024, with the chief complaint of “36 + 6 weeks of pregnancy, vaginal leakage of amniotic fluid for 1 h”. She had regular menstrual cycles, with the last menstruation on February 22, 2024, and the expected date of delivery on November 29, 2024. During regular prenatal examinations in our hospital, she was diagnosed with gestational diabetes (controlled by diet and exercise without medication) and anemia (blood routine showed HGB and MCV lower than normal). Then, 1 h before admission, she developed vaginal amniotic fluid leakage accompanied by irregular abdominal pain and was admitted with the diagnosis of “preterm premature rupture of membranes, *G*4*P*0 + 3, 36 + 6 weeks of intrauterine pregnancy (single fetus in cephalic presentation), and premonitory signs of preterm labor.” Her pre‐pregnancy BMI (body mass index) was 24.7 kg/m^2^, with a weight gain of 9 kg during pregnancy. She had no history of chronic diseases or surgeries, married at 29 years old with three induced abortions, and no special family history.

### 2.2. Obstetrics Examination Upon Admission

With an abdominal circumference of 103 cm, uterine height of 34 cm, and estimated fetal weight of 3300 g, the fetus first appears as the head, the orientation of the fetus in the uterine cavity was LOA (fetal left occipital anterior position), fetal heart rate (HR) of 135 bpm, ruptured membranes, clear amniotic fluid, pH test paper turning blue, and irregular uterine contractions. Auxiliary examinations include the following: blood routine examination (focus on HGB, results detailed in Table 1); fetal ultrasound: fetal head position; BPD (fetal biparietal diameter): 9.5 cm; FL (fetal femur length): 7.4 cm; placenta located on the posterior wall, maturity: Grade I, fetal HR: 135 bpm; and *S*/*D* (the ratio of systolic to diastolic blood flow in fetal cerebral arteries): 1.8. Admission diagnosis includes the following: (1) preterm premature rupture of membranes; (2) diabetes in pregnancy (Grade A1); (3) pregnancy with anemia; and (4) *G*4*P*0 + 3, 36 + 6 weeks intrauterine pregnancy, single fetal head position, and premonitory signs of premature birth.

**Table 1:** Blood routine, coagulation, liver and kidney function, and main blood gas indicators of postpartum women during hospitalization.

**Auxiliary inspection**	**On admission**	**After delivery**	**2 h postop (post 2u RBC transfusion)**	**Postop Day 1**	**Postop Day 2**	**Postop Day 6**
HGB (g/L)	101	97	79	83	75	91
Vaginal drainage volume	—	—	—	20 ml (dark red)	Small amount (light red)	—

Abbreviations: HGB, hemoglobin; RBC, red blood cell.

### 2.3. Diagnosis and Treatment Process

After admission, intravenous oxytocin was given for artificial induction of labor. On November 7, the cervix dilated to 2 cm at 18:00 and fully dilated at 23:00. A male infant (weight 3400 g, length 50 cm) was vaginally delivered in LOA position at 23:39 after lateral episiotomy. The placenta was manually removed 15 min postdelivery. A 2‐cm cervical laceration was sutured immediately; no vaginal wall laceration was found, and perineal incision was sutured. Intrapartum blood loss was ~200 mL; postpartum blood glucose was 5.2 mmol/L, with oxytocin and motherwort used to promote uterine contraction.

Then, 1 h postdelivery, the patient had anal pain, defecation sensation (no bowel movement), dizziness, and fatigue. These symptoms, combined with poor uterine contraction, raised suspicion of potential intrapelvic bleeding. Physical examination: BP (blood pressure) 126/71 mmHg, HR 84 bpm, and poor uterine contraction (improved after 250 *μ*g carboprost tromethamine intramuscular injection). Vaginal examination revealed a left vaginal mass (~10 × 8 *c* *m* ^2^, unclear boundary), which confirmed the diagnosis of vaginal wall hematoma; postpartum hemorrhage warning was activated. A urinary catheter drained ~100 mL pale yellow urine; dual venous access was established, emergency blood routine examination (focus on HGB) was conducted (Table 1), and 6u of type A RH‐positive red blood cell suspension was prepared. It is worth noting that similar nonspecific symptoms (anal pain and defecation urge) have been reported in previous cases of delayed diagnosis of vaginal hematoma, which reminds clinicians to be alert to such atypical manifestations [4].

**Figure 1:**
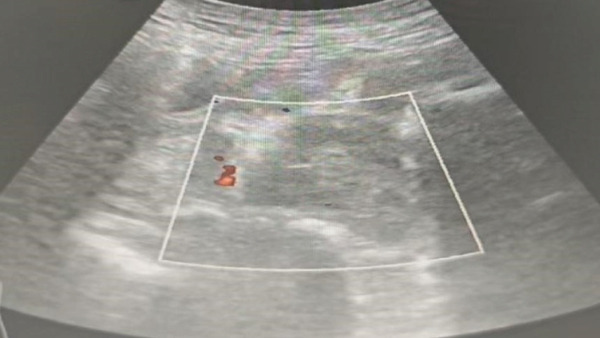
Preoperative ultrasound showing left vaginal wall hematoma.

**Figure 2:**
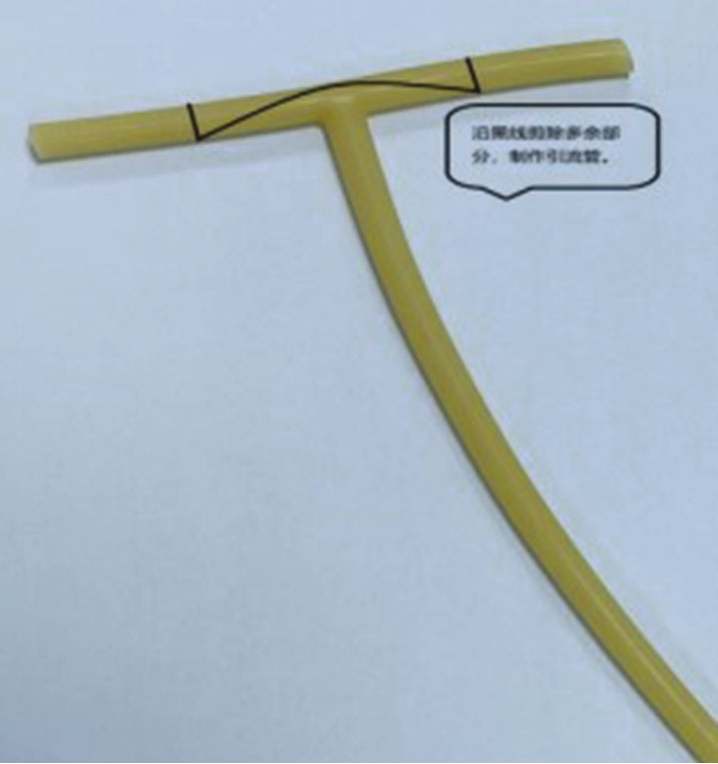
Premodified T‐shaped drainage tube.

**Figure 3:**
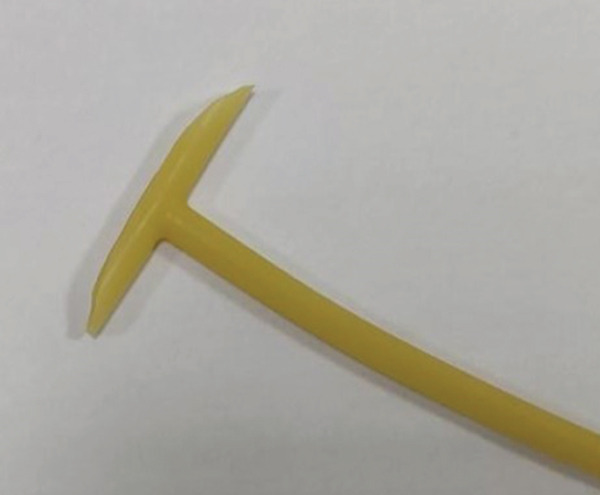
Postmodified T‐shaped drainage tube.

**Figure 4:**
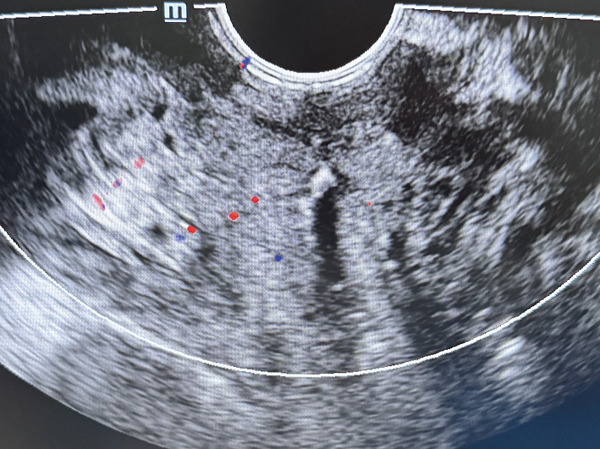
Postoperative ultrasound showing resolution of the hematoma.

At 03:35, under intrathecal block, the perineal suture was removed, and a ‐cm incision was made on the left vaginal wall, draining ~1000 mL blood and clots. Bedside ultrasound showed a left vaginal heterogeneous mass (~5.3 × 4.5 × 5.2 *c* *m* ^3^, clear boundary) (Figure 1), confirming the extent of the hematoma. Studies have confirmed that pelvic ultrasonography is a reliable tool for evaluating the scope of postpartum hematoma, which provides an important basis for formulating surgical plans [5]. Manual exploration removed 500 g clots (cavity extended to pelvic wall); local active bleeding was sutured, but the cavity could not be closed from the bottom. Given the limitations of traditional drainage strips (prone to dislodgment) and single vaginal packing (inadequate drainage)—previous case reports have pointed out that traditional drainage methods often have problems such as inaccurate drainage quantification and easy displacement, which affect the treatment effect [6]—we adopted a combined regimen of “T”‐shaped drainage tube (Figures 2 and 3) and vaginal packing—the T‐type tube ensured stable fixation and quantifiable drainage, while iodine gauze compression prevented rebleeding. The T‐type drainage tube was placed and connected to a drainage bag; two iodine gauze pads were used for vaginal compression. The modification and structure of the T‐shaped drainage tube are shown in Figures 2 and 3: The transverse beam was trimmed to 2 cm in length to ensure stable fixation in the hematoma cavity without tissue irritation. The medical‐grade silicone tube (mucosa‐compatible) was trimmed with sterile scissors; insertion depth was aligned to the hematoma cavity edge (confirmed via intraoperative palpation) for stable fixation. Then, 2 u of red blood cell suspension was transfused intraoperatively. Postoperatively, cefazolin *s* *o* *d* *i* *u* *m* + *m* *e* *t* *r* *o* *n* *i* *d* *a* *z* *o* *l* *e* prevented infection, and oxytocin promoted uterine contraction. The 42‐day postpartum perineal ultrasound showed no residual hematoma or abnormal fluid collection at the original site (Figure 4), indicating complete resolution.

Then, 2 h postoperatively, HGB was 79 g/L (Table 1), and another 2 u of red blood cell suspension was transfused. On Postoperative Day 1, HGB was rechecked (Table 1), with 20 mL of dark red vaginal drainage observed after gauze removal (no active bleeding)—the measurable drainage volume helped confirm no residual massive bleeding. On Postoperative Day 2, only a small amount of light red drainage was noted; the tube was removed (no bleeding), and HGB was rechecked (Table 1)—the minimal drainage indicated effective hemostasis, supporting the decision for early tube removal. On Postoperative Day 6, the patient had no discomfort, with grade A perineal healing and significantly improved HGB compared with previous values (Table 1) and was discharged.

Discharge diagnosis is as follows: (1) severe postpartum hemorrhage; (2) vaginal wall hematoma; (3) preterm premature rupture of membranes; (4) gestational diabetes (Grade A1); (5) pregnancy with *α*‐thalassemia; (6) pregnancy with HPV infection; (7) placental adhesion; (8) vervical laceration; (9) *G*4*P*1 + 3, 36 + 6 weeks pregnancy, vaginal delivery of live male infant; and (10) preterm infant. Then, 10 days postdischarge, follow‐up ultrasound showed closed hematoma cavity and grade A perineal healing.

## 3. Discussion

Postpartum vaginal wall hematoma, especially deep‐seated lesions, is prone to occult bleeding and delayed diagnosis due to its anatomical location, which may progress to severe postpartum hemorrhage if not managed promptly. This case highlights the clinical value of the “T‐type drainage tube + vaginal packing” combined regimen in treating large deep vaginal wall hematoma, and key insights are summarized as follows:

### 3.1. Diagnosis

Deep vaginal wall hematoma is characterized by occult bleeding and nonspecific initial symptoms (e.g., anal pain, defecation sensation, and dizziness) that are easily overlooked [4]. Early diagnosis relies on the combination of postpartum clinical symptoms and targeted vaginal/anal examinations, especially in high‐risk cases (e.g., perineal incisions and anemia) [1]. The patient′s gestational diabetes (may impair tissue healing), pre‐pregnancy anemia (reduces hemoglobin reserve), and multiple abortions (increases soft birth canal fragility) collectively elevated her risk of hematoma formation and progression. In this case, the patient developed typical symptoms 1 h postdelivery, and timely vaginal examination confirmed the hematoma (~10 × 8 *c* *m* ^2^), laying the foundation for effective treatment. The clinical characteristics of this case (occult bleeding and nonspecific symptoms) are consistent with the summary of postpartum hematoma characteristics in previous studies [2], which further verifies the importance of early symptom identification.

### 3.2. Treatment

The selection of the “T‐type drainage tube + vaginal packing” combined regimen was based on the patient′s clinical characteristics: a large hematoma cavity extending to the pelvic wall, with residual space that could not be closed from the bottom after hematoma evacuation. Traditional drainage methods (e.g., drainage strips and single vaginal packing) have limitations such as easy dislodgment, inaccurate drainage quantification, or poor residual blood clearance [6]—a problem also noted in previous reports of concealed paravaginal hematomas, where suboptimal drainage delayed recovery [6]. This contrasts with alternative approaches like Bakri balloon, which is more suited for vaginal–perineal hematomas with uterine bleeding tendencies rather than deep, unclosable cavities [7], and conservative management, which only applies to small, superficial hematomas and risks progression in large lesions [8]. The modified T‐type drainage tube ensures stable fixation and measurable drainage, while iodine gauze packing provides continuous compression to prevent rebleeding. Notably, we did not use tranexamic acid postoperatively; though the drug is recommended for postpartum hemorrhage prevention [9], our patient had stable coagulation, making invasive pharmacologic intervention unnecessary. Additionally, the T‐tube′s trimmed 2‐cm transverse beam aligns with the principle of optimizing drainage devices for postpartum genital hematomas [10], avoiding pelvic tissue irritation while maintaining efficacy—a key improvement over regimens designed for vulvar hematomas [10].

### 3.3. Lessons Learned

Two critical lessons for clinical practice are derived from this case: First, optimizing suturing techniques is essential for preventing hematoma formation. During perineal lateral incision suturing, stitches should start 0.5–1 cm above the wound apex, with sutures perpendicular to the vessel direction to ligate retracted bleeding vessels; overly deep sutures should be avoided to prevent additional injury. Second, strengthening postpartum monitoring is crucial. Obstetric staff should closely observe vital signs and clinical symptoms, and conduct timely vaginal/anal examinations for early detection of hematomas, especially in high‐risk patients. Additionally, effective communication with patients about their condition and treatment plans helps alleviate anxiety and improve treatment compliance.

### 3.4. Patient Perspective

The patient reported sudden anal pain, an unrelieved defecation urge, dizziness, and fatigue 1 h postdelivery, feeling anxious about the symptoms and potential impacts on newborn care. She noted the medical team responded promptly: after examination, the team explained her vaginal wall hematoma and treatment plan (evacuation, T‐type drainage tube placement, and vaginal packing) in plain language, easing her fear. Postoperatively, she mentioned regular nursing checks on vital signs, drainage and wounds, plus guidance on tube care and hygiene‐stating the T‐type tube caused no significant discomfort, and she felt reassured when it was removed on Postop Day 2 without rebleeding. On Postop Day 6, she expressed relief upon learning of Grade A perineal healing and eligibility for discharge. At the 10‐day follow‐up, she reported full recovery and normal newborn care ability and was satisfied with the treatment’s timeliness and effectiveness.

In conclusion, the “T‐type drainage tube + vaginal packing” regimen is safe and effective for large deep vaginal wall hematoma after vaginal delivery, particularly in cases where hematoma cavities cannot be completely closed. It avoids invasive procedures and reduces complication risks, making it worthy of clinical promotion.

## Ethics Statement

This research project has been approved by the Ethics Committee of the First People′s Hospital of Longquanyi District in Chengdu, Sichuan, China (AF‐KY‐2024014).

## Consent

Written informed consent was obtained from the subject and/or guardian.

## Disclosure

Funding details are for transparency, funds did not affect study processes or manuscript writing.

This case report complies with the CARE Consensus. The included supporting information “CARE Checklist_4448042.docx” provides the completed CARE Checklist, meeting the journal’s reporting requirements.

## Conflicts of Interest

The authors declare no conflicts of interest.

## Funding

This work was supported by the Traditional Chinese Medicine Research Special Project of Sichuan Provincial Administration of Traditional Chinese Medicine in China, No. 2024MS306.

## Supporting information


**Supporting Information** Additional supporting information can be found online in the Supporting Information section.

## Data Availability

The data that support the findings of this study are available on request from the corresponding author. The data are not publicly available due to privacy or ethical restrictions.
