# Supplementary material for: Application of T‐Type Drainage Tube in Treating Large Deep Vaginal Hematoma Postpartum: A Technical Note
Source: Case Rep Obstet Gynecol. 2026 Jan 30;2026:4448042. doi: 10.1155/crog/4448042 (PMC12857459; doi:10.1155/crog/4448042)
Supplement: Supplementary file 1 — Supporting Information Additional supporting information can be found online in the Supporting Information section. [file CROG-2026-4448042-s001.docx]

**CARE Checklist – Case Report**

**Title:** Application of T-type Drainage Tube in Treating Large Deep Vaginal Hematoma Postpartum: A Technical Note

**Key Words:** Vaginal delivery; Deep vaginal wall hematoma; T-shaped drainage catheter; Vaginal packing; Case report

**Abstract (no references)**

**3a.** Introduction: What is unique about this case and what does it add to the scientific literature?

This case report presents the innovative application of a modified “T-type drainage tube combined with vaginal packing” regimen for managing a large, deep postpartum vaginal wall hematoma that could not be surgically closed. It provides a safe, effective, and minimally invasive technical alternative to traditional drainage methods or open abdominal surgery for this challenging complication, adding to the limited literature on optimized drainage techniques for deep pelvic hematomas.

**3b.** Main symptoms and/or important clinical findings

One hour after an uncomplicated vaginal delivery, the patient developed anal pain, a persistent sensation of needing to defecate (tenesmus), dizziness, and fatigue. Physical examination revealed a large, firm, and tender mass (~10×8 cm²) on the left vaginal wall. Bedside transvaginal ultrasound confirmed a heterogeneous hematoma measuring approximately 5.3×4.5×5.2 cm³.

**3c.** The main diagnoses, therapeutic interventions, and outcomes

Main Diagnoses: 1) Large deep postpartum vaginal wall hematoma; 2) Severe postpartum hemorrhage.

Interventions: Surgical evacuation of the hematoma (~1000 mL blood and 500g clots) followed by placement of a modified medical-grade silicone T-type drainage tube (transverse arm trimmed to 2cm) and vaginal packing with iodine gauze. Prophylactic antibiotics (cefazolin sodium + metronidazole) and blood transfusion (4 units of RBCs) were administered.

Outcomes: The drainage tube was removed on postoperative day 2 as drainage diminished. The patient was discharged on postoperative day 6 with well-healed perineal wounds (Grade A). Follow-up ultrasound at approximately 42 days postpartum showed complete resolution of the hematoma cavity.

**3d.** Conclusion—What is the main “take-away” lesson(s) from this case?

For large, deep vaginal wall hematomas where the cavity cannot be primarily closed, the combined regimen of a modified T-type drainage tube and vaginal packing is a safe and effective treatment. It ensures quantifiable drainage, stable fixation, and continuous compression, facilitating rapid recovery and avoiding more invasive procedures. Early recognition of non-specific symptoms like postpartum tenesmus and anal pain is crucial for timely diagnosis.

**Introduction**

The introduction section of the manuscript fulfills this requirement.

**Patient Information**

**5a.** De-identified patient specific information.

A 30-year-old female (Gravida 4, Para 0+3).

**5b.** Primary concerns and symptoms of the patient.

On admission: “Vaginal leakage of amniotic fluid for 1 hour” at 36+6 weeks gestation.

Postpartum (1 hour after delivery): Sudden onset of severe anal pain, an unrelieved urge to defecate (tenesmus), dizziness, and fatigue.

**5c.** Medical, family, and psycho-social history including relevant genetic information.

Medical History: Gestational diabetes mellitus (A1, diet-controlled). Pre-pregnancy anemia (suspected α-thalassemia trait based on low HGB and MCV). History of HPV infection. Three prior induced abortions. No chronic hypertension, coagulation disorders, or previous vaginal surgery. No known drug allergies.

Obstetric History: G4P0+3. Regular prenatal care.

Family History: No special family history reported.

Social History: Married. Pre-pregnancy BMI: 24.7 kg/m².

**5d.** Relevant past interventions with outcomes.

The patient had no relevant past surgical interventions or medical treatments for similar conditions.

**Clinical Findings**

**6.** Describe significant physical examination (PE) and important clinical findings.

Postpartum PE (1 hour after delivery): Vital signs: Blood pressure 126/71 mmHg, Heart rate 84 bpm. Uterine contraction was poor initially but improved after uterotonic administration.

Focused Vaginal Examination: Revealed a large, firm, and tender mass, approximately 10×8 cm², with unclear boundaries, located on the left vaginal wall. Significant perineal swelling was noted.

**Timeline**

**7.** Historical and current information from this episode of care organized as a timeline

2024-11-07 (Admission): Admitted with preterm premature rupture of membranes. Labor induced with oxytocin.

2024-11-07, 23:39: Vaginal delivery of a live male infant (3400g) with lateral episiotomy. Estimated blood loss ~200mL.

~1 hour Post-delivery: Patient reported anal pain, tenesmus, dizziness. Vaginal exam confirmed a large hematoma. Postpartum hemorrhage protocol activated.

2024-11-08, ~03:35 (Approx. 4 hours post-delivery): Under intrathecal block anesthesia, hematoma was surgically evacuated, and a modified T-type drainage tube with vaginal packing was placed.

Postoperative Day 1: Drainage fluid was 20ml dark red. Vaginal packing removed.

Postoperative Day 2: Drainage minimal (light red). T-type drainage tube removed.

Postoperative Day 6: Patient asymptomatic, perineal wound healed well (Grade A). Discharged from hospital.

~10 days Post-discharge (~42 days postpartum): Follow-up transperineal ultrasound showed complete resolution of the hematoma cavity.

**Diagnostic Assessment**

**8a.** Diagnostic testing (such as PE, laboratory testing, imaging, surveys).

Physical Examination: As detailed in item 6.

Laboratory Testing: Serial blood routine tests (HGB levels: 101 g/L on admission, 97 g/L post-delivery, 79 g/L 2 hours post-op, 83 g/L on POD1, 75 g/L on POD2, 91 g/L on POD6).

Imaging: Bedside transvaginal ultrasound performed post-delivery confirmed a left vaginal wall heterogeneous mass (~5.3×4.5×5.2 cm³) consistent with a hematoma (Figure 1). Follow-up ultrasound at 42 days postpartum showed complete resolution (Figure 4).

**8b.** Diagnostic challenges (such as access to testing, financial, or cultural).

No significant financial or cultural barriers to diagnosis were reported. The primary diagnostic challenge was the atypical and non-specific nature of the initial symptoms (anal pain and tenesmus rather than overt vaginal bleeding or severe pain), which could lead to delayed recognition if a high index of suspicion is not maintained.

**8c.** Diagnosis (including other diagnoses considered).

Primary Diagnosis: Large deep postpartum vaginal wall hematoma; Severe postpartum hemorrhage.

Differential Diagnoses Considered: Other causes of postpartum pelvic pain and bleeding were considered and ruled out by examination and ultrasound, such as uterine atony, retained placental fragments, or broad ligament hematoma. The focal vaginal mass on exam and ultrasound localized the problem to the vaginal wall.

**8d.** Prognosis (such as staging in oncology) where applicable.

With timely and effective surgical drainage and hemostasis, the prognosis for postpartum vaginal hematoma is excellent for full recovery without long-term sequelae. This patient’s prompt treatment led to complete anatomical resolution and functional recovery, as confirmed by follow-up.

**Therapeutic Intervention**

**9a.** Types of therapeutic intervention (such as pharmacologic, surgical, preventive, self-care).

Surgical: Incision and evacuation of hematoma; Placement of modified T-type silicone drainage tube; Vaginal packing with iodine gauze; Suturing of active bleeding points.

Pharmacologic: Prophylactic antibiotics (Cefazolin sodium + Metronidazole); Uterotonics (oxytocin, carboprost tromethamine); Intravenous fluid resuscitation; Blood transfusion (4 units of A+ RBCs).

Supportive/Nursing Care: Postoperative monitoring of vital signs and drainage; Daily perineal care with povidone-iodine; Drainage tube care and output recording; Patient education on hygiene and activity.

**9b.** Administration of therapeutic intervention (such as dosage, strength, duration).

Surgery: Performed under intrathecal block anesthesia. The transverse arm of the T-tube was trimmed to 2cm before placement.

Antibiotics: Administered postoperatively for infection prophylaxis. Specific dosage/duration detailed in the manuscript’s methods/care plan.

Blood Transfusion: A total of 4 units of A Rh-positive red blood cell suspension were transfused (2 units intraoperatively, 2 units postoperatively).

Drainage Tube: Removed on Postoperative Day 2 when 24-hour drainage was minimal.

**9c.** Changes in therapeutic intervention (with rationale).

The treatment plan was executed as decided upon diagnosis. There was no mid-course change in the core surgical or pharmacologic regimen. The decision to use the T-tube + packing combination was made intraoperatively after assessing that the hematoma cavity extended to the pelvic wall and could not be closed from the bottom, making traditional simple drainage or packing alone suboptimal.

**Follow-up and Outcomes**

**10a.** Clinician and patient-assessed outcomes (if available).

Clinician-assessed: Successful hemostasis, resolution of hematoma on ultrasound (Figure 4), Grade A perineal wound healing, normalization of hemoglobin trend, and uncomplicated discharge.

Patient-assessed: The patient reported resolution of symptoms, no significant discomfort from the drainage tube, satisfaction with the explanation of care, and relief at the rapid recovery, enabling her to resume newborn care. (Detailed in the “Patient Perspective” section of the manuscript).

**10b.** Important follow-up diagnostic and other test results.

Follow-up transperineal ultrasound performed approximately 42 days postpartum showed a closed hematoma cavity with no residual fluid collection or hematoma at the original site.

**10c.** Intervention adherence and tolerability (How was this assessed?).

Adherence & Tolerability: The patient adhered well to postoperative care instructions regarding ambulation and perineal hygiene. She tolerated the T-type drainage tube well, reporting no significant pain or discomfort from it.

Assessment Method: Adherence and tolerability were assessed through daily nursing checks, direct patient questioning during rounds about comfort and understanding of care instructions, and observation of the drainage site and tube fixation.

**10d.** Adverse and unanticipated events.

No adverse or unanticipated events related to the surgical procedure, T-tube placement, or postoperative course were reported.

**Discussion**

The manuscript contains a comprehensive "Discussion" section that addresses the strengths, limitations, literature review, rationale, and take-away lessons of this case.

**Patient Perspective**

**12.** The patient should share their perspective in one to two paragraphs on the treatment(s) they received.

The manuscript includes a dedicated "Patient Perspective" subsection (2.4), detailing the patient's experience from symptom onset through recovery and her satisfaction with the treatment process.

**Informed Consent**

**13.** Did the patient give informed consent?

Yes. Written informed consent was obtained from the patient for the publication of this case report and any accompanying images. All patient-identifying information has been de-identified.
